# Handheld endomicroscope using a fiber-optic harmonograph enables real-time and in vivo confocal imaging of living cell morphology and capillary perfusion

**DOI:** 10.1038/s41378-020-00182-6

**Published:** 2020-09-21

**Authors:** Kyungmin Hwang, Yeong-Hyeon Seo, Daniel Y. Kim, Jinhyo Ahn, Soyoung Lee, Kyung Hee Han, Koun-Hee Lee, Sangyong Jon, Pilhan Kim, Kate E. Yu, Hyungsin Kim, Shin-Hyuk Kang, Ki-Hun Jeong

**Affiliations:** 1 Department of Bio and Brain Engineering, KAIST and KAIST Institute of Health Science and Technology, Daejeon, 34141 Republic of Korea; 2 Graduate School of Nanoscience and Technology, KAIST and KAIST Institute of Health Science and Technology, Daejeon, 34141 Republic of Korea; 3 Department of Biological Sciences, KAIST and KAIST Institute for the BioCentury, Daejeon, 34141 Republic of Korea; 4 Hyunjoo In-Tech, Seoul, 08390 Republic of Korea; 5 VPIX Medical, Inc, Deajeon, 34141 Republic of Korea; 6 Graduate School of Medical Science and Engineering, Daejeon, 34141 Republic of Korea; 7 Department of Neurosurgery, Korea University Anam Hospital, Korea University Medicine, Seoul, 02842 Korea

**Keywords:** Optical sensors, Electrical and electronic engineering, Environmental, health and safety issues

## Abstract

Confocal laser endomicroscopy provides high potential for noninvasive and in vivo optical biopsy at the cellular level. Here, we report a fully packaged handheld confocal endomicroscopic system for real-time, high-resolution, and in vivo cellular imaging using a Lissajous scanning fiber-optic harmonograph. The endomicroscopic system features an endomicroscopic probe with a fiber-optic harmonograph, a confocal microscope unit, and an image signal processor. The fiber-optic harmonograph contains a single mode fiber coupled with a quadrupole piezoelectric tube, which resonantly scans both axes at ~ 1 kHz to obtain a Lissajous pattern. The fiber-optic harmonograph was fully packaged into an endomicroscopic probe with an objective lens. The endomicroscopic probe was hygienically packaged for waterproofing and disinfection of medical instruments within a 2.6-mm outer diameter stainless tube capable of being inserted through the working channel of a clinical endoscope. The probe was further combined with the confocal microscope unit for indocyanine green imaging and the image signal processor for high frame rate and high density Lissajous scanning. The signal processing unit delivers driving signals for probe actuation and reconstructs confocal images using the auto phase matching process of Lissajous fiber scanners. The confocal endomicroscopic system was used to successfully obtain human in vitro fluorescent images and real-time ex vivo and in vivo fluorescent images of the living cell morphology and capillary perfusion inside a single mouse.

## Obtaining penetrating insights into in vivo biology

A fiber-optic microscopy system collects real-time imaging data with high resolution from within the tissue of living organisms. Endoscopically-coupled microscopes systems have become increasingly commonplace, but these systems often suffer from stability issues and trade-offs in terms of imaging speed and resolution. Researchers led by Ki-Hun Jeong of the KAIST Institute of Health Science and Technology have now developed an endomicroscope based on a system known as a Lissajous fiber scanner, a design that delivers excellent stability and uniform image quality. The researchers demonstrate the capacity to obtain micron-resolution imaging data from cultured cells, isolated organs, and live mice at a rate of 10 frames per second. The device is compact and designed for handheld use, and the researchers already envision opportunities to further improve imaging depth and resolution in the near future.

## Introduction

In vivo microscopic techniques provide many opportunities for a better understanding of various biomedical interactions, such as cell-microenvironment interactions, molecular expression, or cell infiltration^1–3^. However, conventional optical microscopes still have some technical limitations in imaging through the skin surface or a surgical incision due to tissue light transmission limitations, large objective lens, and sample stage fixation^4^. Minimized laser scanning microscopes using a microscanner and a scanning optical fiber serve as an enabling tool and have rapidly led to diverse biological or clinical applications^5–8^, including mounting an endomicroscope on the head of a mouse^9^. Recently, endomicroscopes have been fit into the working channel of a conventional endoscope, and the clinical feasibility for optical biopsy has been assessed^10^. Optical biopsy has become a new paradigm for cancer diagnosis^10^ beyond the clinical limitations of conventional biopsy, such as the requirement for tissue extraction, critical time-consuming procedures, and tissue sampling errors^6^. In addition, clinical trials have been actively performed with commercialized endomicroscopes based on proximal scanning with the fiber bundle^11^. However, compact packaged endomicroscopic systems are still challenging, particularly for high-resolution imaging with a large field of view. More recently, assorted microscanners have been integrated at the distal end of an optical fiber for high-resolution endomicroscopic imaging.

Microscanners, such as scanning mirrors^12–14^, scanning lenses^15^, or scanning fiber operation systems^16–18^, actively allow laser scanning endomicroscopic applications. Scanning MEMS mirrors offer high design flexibility, but there are still some fundamental limitations to compact packaging for en face endomicroscopic imaging applications due to beam deflection^19^. Scanning MEMS lenses also have intrinsic limitations in achieving a fast scanning speed for real-time imaging due to the lens inertia^20^. Electrostatic^13^, electromagnetic^14^, or electrothermal^17^ MEMS actuation may raise further technical barriers, such as poor scanner stability, high driving voltages or currents, or low scanning speed^5^. In contrast, scanning fibers actuated by a piezoelectric tube (PZT) with quadrupole electrodes are very beneficial in terms of both compact packaging and high mechanical stability. A single bare fiber scanning at resonance along orthogonal axes readily forms a spiral pattern of the laser beam due to the circular cross-section^21^. However, the spiral scan exhibits a high illumination density in the center area, which may result in photobleaching or photodamage^22^. In addition, undesirable small off-axis misalignment between the PZT tube and a single fiber often causes substantial distortion of spiral patterns due to the cross-talk between the orthogonal scanning axes^23^. Recently, Lissajous scanning has been applied to the fiber scanner by attaching an additional silicon microstructure to asymmetrically modulate the stiffness of a single optical fiber^16^. A Lissajous fiber scanner often resonates at two different high scanning frequencies due to the high spring constants for both orthogonal axes, which provide high mechanical stability. In addition, Lissajous figures are more uniform in the central area than spiral patterns. However, conventional Lissajous scanning is still challenging due to the manufacturing tolerance, environmental changes, and the complex relationship among the stiffness, deflection force, and damping factors of both axes^24^. In particular, Lissajous fiber scanners often have a trade-off between the scanning speed and the scanning density, which restrains the use of Lissajous microscanners for high-speed and high-resolution imaging applications^25^. Recently, the frequency selection rule has enabled high definition and high frame rate (HDHF) Lissajous scanning, considering the greatest common divisor (GCD) between the frequencies and the total lobe number, which is the sum of the scanning frequencies divided by the GCD^26^. HDHF Lissajous scanning is performed at the selected scanning frequencies, where the total lobe number satisfies the target high fill factor and the GCD is the maximum, substantially overcoming the disadvantages of conventional Lissajous scanners.

**Fig. 1:**
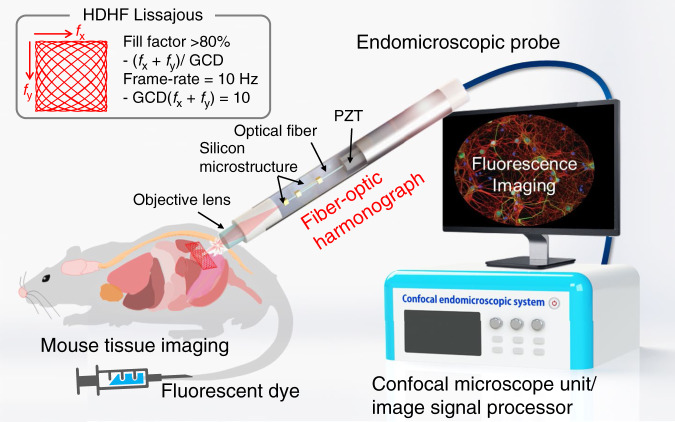
Schematic illustration of a fully packaged confocal endomicroscopic system for real-time and in vivo cellular imaging using a fiber-optic harmonograph. The system configuration comprises an endomicroscopic probe, a confocal microscope unit, and an image signal processor. The fiber-optic harmonograph inside the endomicroscopic probe resonates a single mode fiber at ~1kHz, based on the HDHF frequency selection rule. The endomicroscopic probe is then combined with an indocyanine green (ICG) fluorescence confocal microscope unit and a phase-correctable image signal processor (ISP)

Here, we report a fully packaged confocal endomicroscopic system for real-time, high-resolution, and in vivo cellular imaging using a fiber-optic harmonograph, i.e., an HDHF Lissajous fiber scanner. A harmonograph, invented in the mid-19th century, serves as a geometric drawing tool for miscellaneous Lissajous figures by changing the frequency and phase of two oscillating pendulums in perpendicular directions. The optical analog was realized by an HDHF Lissajous scanning fiber and fully developed at a standalone endomicroscopic system level for fast in vivo cellular and microvascular imaging applications. The endomicroscopic system comprises an endomicroscopic probe, a confocal microscope unit, and an image signal processor (ISP) (Fig. 1). The endomicroscopic probe consists of an objective lens and the fiber-optic harmonograph analog to the conventional harmonograph, which is based on a miniaturized actuator with an optical fiber. A single-mode optical fiber inside the fiber-optic harmonograph resonates at ~1 kHz, based on the frequency selection rule for HDHF imaging. As a result, the scanning pattern exhibits a frame rate of 10 Hz and a fill factor of over 80%, corresponding to a GCD of 10 for both axis scanning frequencies of *f*_x_ and *f*_y_ and a total lobe number [(*f*_x_ + *f*_y_)/GCD] of over 203. The endomicroscopic probe is also coupled with a portable confocal microscope unit for indocyanine green (ICG) fluorescence imaging and further with the ISP for phase-correctable image reconstruction. The fully integrated Lissajous scanning confocal endomicroscope enables real-time in vivo cellular and tissue imaging for fluorescently labeled small animals.

## Results

**Fig. 2:**
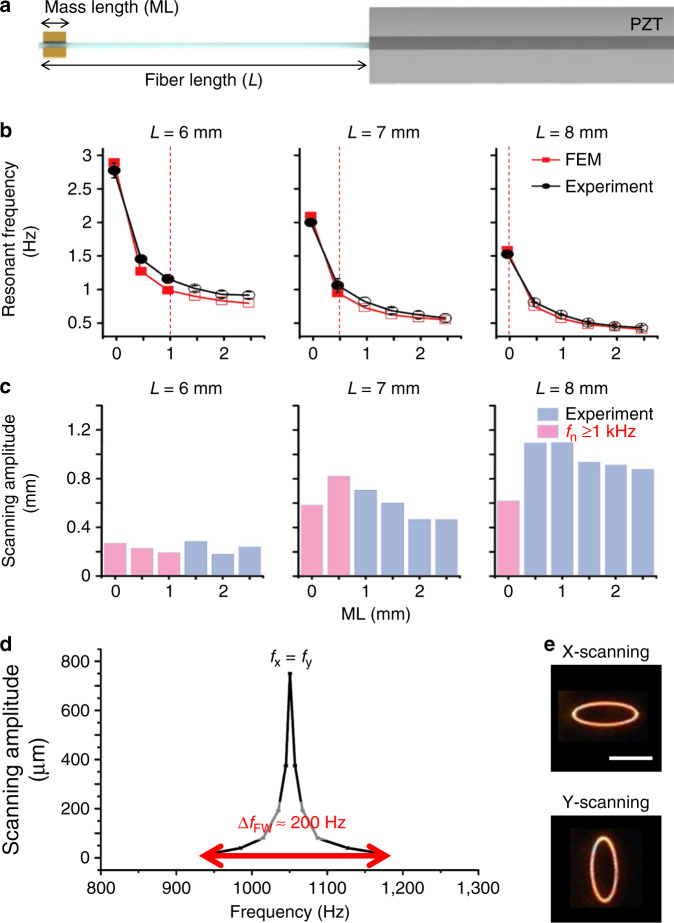
Resonant frequency analysis of the fiber scanner before attaching a silicon spring. **a** Schematic of the fiber scanner with the silicon mass structure. The fiber length (*L*) and the silicon mass length (ML) mainly determine the resonant frequencies. **b** Calculated and measured resonant frequencies depending on *L* and ML. The *L* and ML for over 1-kHz resonant frequency are displayed as filled circles. **c** Scanning amplitude of a resonating fiber tip. The *L* and ML for over 1-kHz resonant frequency are colored in pink. **d** Frequency response of the fiber scanner for L = 7 mm and ML = 0.5 mm. The resonant frequency is 1050Hz with a Q-factor of 82 and a full-width scanning bandwidth of 200 Hz. **e** 1D scanning lines showing mechanical cross-coupling. Scale bar = 500 μm

**Fig. 3:**
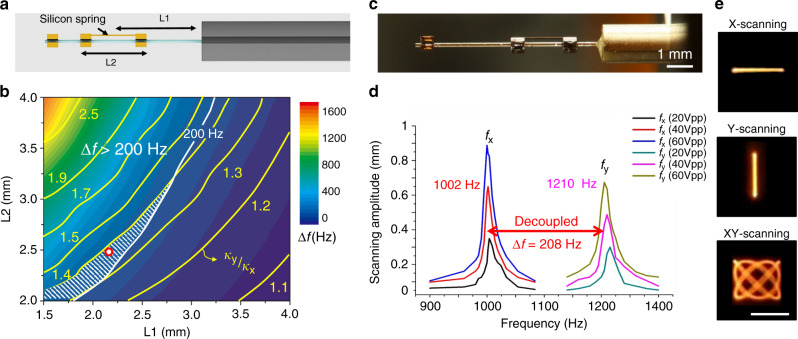
Frequency separation of the fiber-optic harmonograph for high-definition and high frame rate Lissajous scanning. **a** Schematic illustration of the fiber-optic harmonograph with a silicon spring and mass. **b** Color map from FEM analysis of ∆*f* depending on *L*1 and *L*2. The yellow contour lines of *κ*_y_/*κ*_x_ depending on *L*1 and *L*2 are placed over the ∆*f* color map. *L*1 and the *L*2 were set to be 2.25 mm and 2.5 mm within the red region (∆*f***>** 200 Hz and *κ*_y_/*κ*_x_**<** 1.4). **c** Optical image of the fiber-optic harmonograph consisting of a quadruple PZT tube, a single mode optical fiber, and micromachined silicon structures. **d** Frequency response of the fiber-optic harmonograph. The resonant frequencies for the *x*-axis and *y*-axis are fully decoupled without any mechanical coupling. **e** 1D scanning lines showing clear line patterns without mechanical cross-coupling. Scale bar = 500 μm

The fiber-optic harmonograph comprises a PZT tube (PIC151, PI Korea Ltd.) with quadrupole electrodes, a single-mode optical fiber, micromachined silicon structures for modulating the inertia of the fiber tip and spring constants for both axes. The resonant frequency of a scanning fiber (*f*_r_) is governed by *f*_r_ = 1/2*π*√(*κ*/*m*_eff_), where *κ* and *m*_eff_ are the spring constant and the effective mass, respectively^27^. The fiber length (*L*) and the silicon mass (volume: 500 μm × 400 μm × length) length (ML) modulate the resonant frequencies for both axes (Fig. 2a), which were analyzed by using finite element analysis (FEA) and an experiment (Fig. 2b). The resonant frequency increases with decreasing fiber length or mass length. *L* and ML for resonant frequencies over 1 kHz are displayed as filled circles. The scanning amplitudes of a scanning fiber at resonance depending on *L* and ML were experimentally obtained (Fig. 2c). The scanning amplitudes for *L* = 6 mm have irregular results with ML due to the high stiffness and a low Q-factor. The scanning amplitudes for *L* = 7 and 8 mm increase and decrease with ML due to high air damping and large inertia. *L* and ML are shown in pink for resonant frequencies over 1 kHz. Finally, the fiber length *L* and the mass length ML were designed to be 7 mm and 0.5 mm, respectively, which resulted in both a resonant frequency of ~1 kHz and the maximum scanning amplitude. In the experiment, the fiber scanner shows a resonant frequency of 1050 Hz and a full width (Δ*f*_FW_) of 200 Hz (Fig. 2d). A scanning fiber with a circular cross-section often exhibits unfavorable mechanical coupling for the two axes due to the same resonant frequencies of the *x*-axis and *y*-axis, as shown in Fig. 2e. The asymmetric attachment of a micromachined spring effectively differentiates the biaxial spring constants of a single fiber over the bandwidth and thus results in complete separation of the resonant frequencies of the two axes (∆*f*). The silicon spring location (*L*1), which is the distance from the PZT to the center of gravity of the spring, and the silicon spring length (*L*2) in Fig. 3a directly determine the frequency separation ∆*f* and the biaxial stiffness ratio (*κ*_y_/*κ*_x_). ∆*f* and *κ*_y_/*κ*_x_ depending on *L*1 and *L*2 were calculated by using FEA and are expressed in a color map and contour lines (Fig. 3b). ∆*f* and *κ*_y_/*κ*_x_ increase as *L*1 shortens and *L*2 lengthens; however, as *L*1 becomes long, the silicon spring may act as an additional silicon mass rather than as an asymmetric spring structure and thus reduce the resonant frequency of a scanning fiber. Both *κ*_y_/*κ*_x_ and Δ*f* increase as *L*2 increases. For this experiment, Δ*f*, i.e., *f*_y_−*f*_x_, should be over Δ*f*_FW_ (200 Hz) to eliminate the cross-coupling, and *κ*_y_/*κ*_x_ should be close to one to obtain a similar scanning amplitude for both axes. As a result, *L*1 was set to 2.25 mm, and *L*2 was set to 2.5 mm. The fabricated fiber-optic harmonograph (Fig. 3c) shows resonant frequencies of 1002 Hz for the *x*-axis and 1210 Hz for the *y*-axis without any mechanical coupling (Fig. 3d, e). The driving frequencies for the fabricated fiber-optic harmonograph were set to be 1000 Hz and 1210 Hz for each axis to obtain a 10 frame rate and an over 80% fill factor according to the HDHF frequency selection rule.

**Fig. 4:**
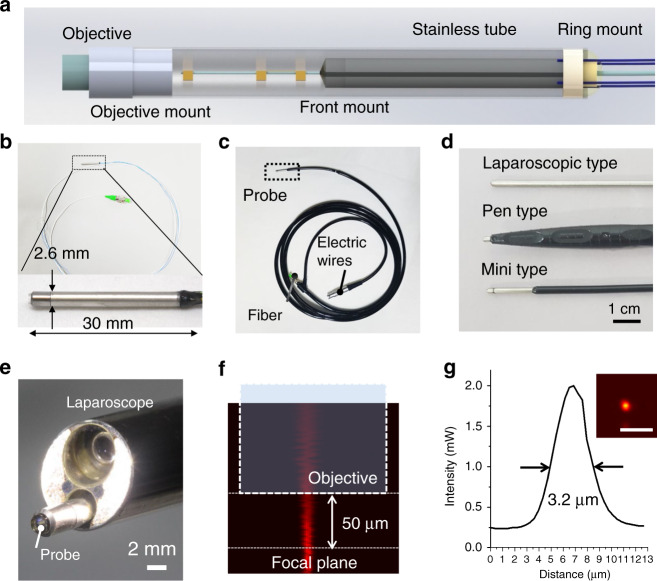
Endomicroscopic probe. **a** Schematic illustration of the fully packaged fiber-optic harmonograph, including an objective mount, front/ring mounts, and a fiber scanner. **b** Fully packaged fiber-optic harmonograph (OD = 2.6 mm, length = 30mm). **c** Endomicroscopic probe packaged for waterproofing and sterilization. **d** Three different packages of the laparoscopic (OD = 3.4mm, length = 300mm), pen, and mini (OD = 2.6 mm, length = 30mm) types. **e** Laparoscopic type fitting into the working channel of a conventional laparoscope. **f** The working distance of 50 μm was measured by optical sectioning along the *z*-axis with a conventional confocal microscope. **g** Beam profile at the focal plane. The lateral resolution is 3.2 μm. Scale bars are 10 μm

The fiber scanner was successfully assembled with micromachined silicon microstructures. The fiber-optic harmonograph was precisely assembled with a customized objective lens (NEM-180-25-10-860-S, GRINTECH GmbH), a lens mount, a front mount, and a ring mount (Fig. 4a). The diameter of the lens was set to 1.8 mm so that it could be packaged within a 2.6-mm outer diameter probe, and the working distance of the tissue side was set to 100 µm to make the probe contact type for handheld imaging. The ring mount effectively helps precise alignment between the PZT tube and housing tube, and the front mount facilitates centering of a single fiber inside the PZT tube. All the mounts were fabricated using a 3D printer (Projet3500, 3D systems). Probe packaging was performed manually using a microscope. For more precise packaging, after packaging using the mounts, UV curable resin (NOA 61 and 63, Thorlabs Inc) was applied to the connective point between the mounts and fixed by UV exposure while microscopically verifying that the mounts had their center axes aligned. The physical dimensions of the fully packaged fiber-optic harmonograph are 2.6 mm in diameter and 30 mm in length (Fig. 4b). The endomicroscopic probe was fully packaged with waterproofing and autoclaved sterilization for three different endomicroscopic probes of the laparoscopic, pen, and mini types (Fig. 4c, d).

The laparoscopic probe is 3.4 mm in OD, which is readily inserted into the accessory channel of a conventional laparoscope (Fig. 4e). The pen type also serves as a handheld confocal microscope, and the mini type of 2.6 mm in outer diameter is utilized for noninvasive or minimally invasive endomicroscopic applications. The probe is designed as a contact probe with a working distance of <100 μm. The measured working distance and the beam spot size are 50 μm in air and 3.2 μm, respectively (Fig. 4f, g).

**Fig. 5:**
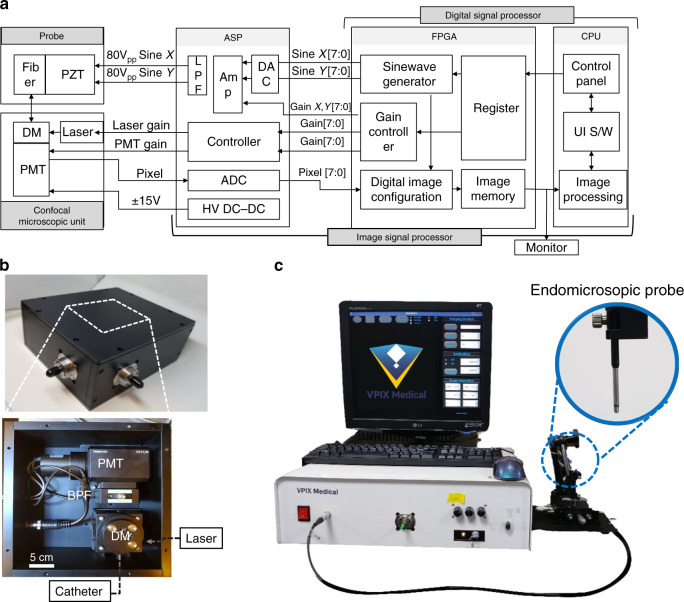
Confocal endomicroscopic system using a fiber-optic harmonograph. **a** block diagram of the confocal endomicroscopic system including an endomicroscopic probe, a confocal microscope unit, and an image signal processor (ISP). The ISP includes an analog signal processor (ASP) and a digital signal processor (DSP) for reconstruction of confocal images. **b** Top-side view of the confocal microscope unit including a fiber-coupled laser (785 nm, LASOS785LDM), DM dichroic mirror, PMT photomultiplier, BPF bandpass filter. **c** Fully developed confocal endomicroscopic system

The endomicroscopic probe was finally combined with a home-built confocal microscopic system including the confocal microscope unit and the ISP (Fig. 5a). The probe has a fully repeating pattern frame of 10 Hz with a fill rate of over 87% using the HDHF selection rule. The confocal microscope unit includes a light source laser (LASOS785LDM, 785 nm, 50 mW), a dichroic mirror (DM), a bandpass filter (BPF), and a photomultiplier tube (PMT). Optical alignment between the excitation laser and the endomicroscopic probe was performed using two achromatic fiber ports (PAFA-X-4-B, Thorlabs, Inc.). The dichroic mirror reflects a laser beam and transmits fluorescence signals through the endomicroscopic probe, which are finally collected by the PMT after the BPF (Fig. 5b). The ISP includes an analog signal processor (ASP) and a digital signal processor (DSP) for reconstruction of confocal images. The ASP includes driving voltage outputs for PZT actuation, an analog-to-digital converter (ADC) for input pixel signals, and controllers for controlling the laser power and the PMT gain in the confocal microscope unit to prevent photobleaching or photodamage, respectively. The driving voltage outputs, which are two different sine waves of ~1 kHz under 80 V_pp_, are generated using a look-up table by a sine wave generator in the DSP with 8-bit resolution. The driving voltage signals are amplified and passed through the low-pass filter to actuate the PZT. The signals are also converted to the position coordinates of confocal images. The DSP also receives the PMT signal through the ADC using a 10-M sampling clock with 8-bit resolution and finally reconstructs it into a confocal image of 256 × 256 pixels on the field programmable gate array (FPGA) board integrated with a simple median filter for unscanned pixels. The image memory in the FPGA board displays the image using the pixel update algorithm, which sends pixel data every 1/60 s to the monitor instead of sending the image data every actual frame (0.1 s). The algorithm allows some of the whole image to be updated faster than the original probe’s frame rate of 0.1 s. The DSP also enables a phase calibration process using the standard deviation of the signals coming into the image pixel to determine the phase matching and calculate the phase delay. Figure 5c shows the fully developed confocal endomicroscopic system.

**Fig. 6:**
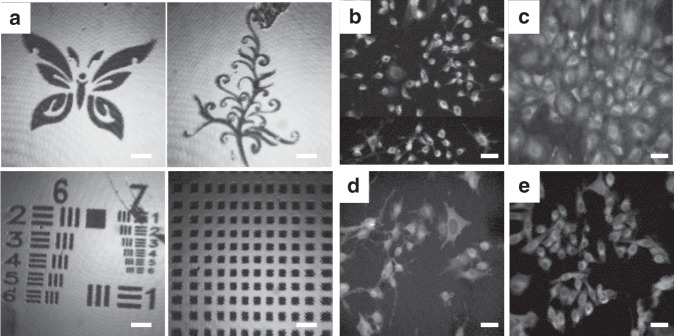
Confocal test images. **a** 2D reflectance imaging results of various metal patterns. (**b**–**e**) Cellular fluorescence imaging results. ICG was fed to various cell lines for one hour. (**b**) Colon carcinoma (CT 26). (**c**) Human lung cancer (A549 cells). (**d**) LNcaP prostate. (**e**) Human oral cancer (KB Oral). Laser power = 2 mW

**Fig. 7:**
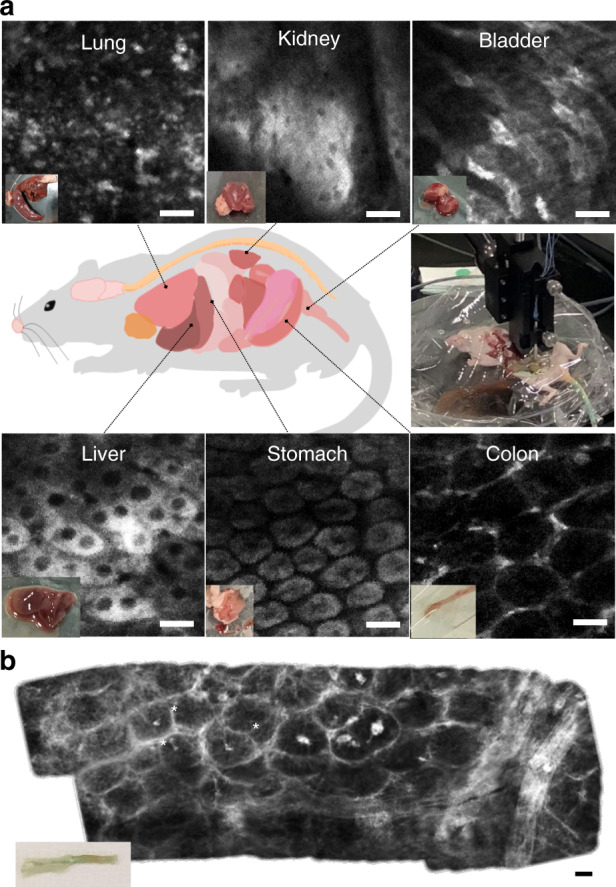
Ex vivo mouse organ imaging results of a mouse after ICG perfusion surgery. **a** Imaging results of the lung, kidney, bladder, duodenum, colon, stomach, and liver without any post sample preparation process. **b** Stitched result of individual images of the colon. The historical morphology of the colon could be distinguished such as crypt structures surrounding blood vessels looking like a honeycomb and polyps (asterisks in the figures) in every crypt. The white areas show ICG signals, and the black area in a crypt is likely to be a nucleus. Scale bar = 20 μm

**Fig. 8:**
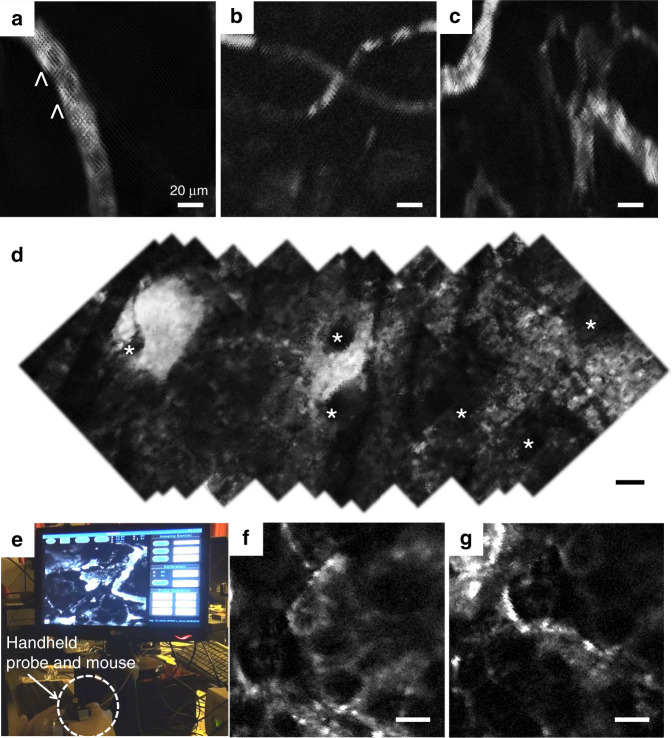
Confocal in vivo images of an anesthetized mouse beneath the laparoscopic-type probe. The probe obtained real-time imaging results while in contact with the target tissue. **a**–**c** Fluorescence images of the blood vessel from the bladder. ICG molecules inside the blood vessel were imaged (Visualizations 1, 2, and 3). **d** Stitched fluorescence image of dermal tissue in the ear skin. Hair follicles (asterisks) were observed. **e** Mouse skin in vivo imaging process with the handheld endomicroscopic probe (Visualization 4). **f**, **g** Fluorescence image of dermal tissue of the nude mouse after topically applying ICG on the skin. Adipose tissue is observed as a black space surrounded by a polygonal fluorescent line. Scale bars are 35 μm

The endomicroscopic system was used to successfully obtain various confocal reflectance images of metal patterns, including an industry standard United States Air Force (USAF) Resolving Power 1951 test pattern (Fig. 6a), in vitro fluorescence images (Fig. 6b), ex vivo mouse fluorescence images (Fig. 7), and in vivo mouse fluorescence images (Fig. 8), by using the handheld probe. The FOV is 350 μm × 350 μm at a driving voltage of 20 V_pp_.

## Discussion

The fiber-optic harmonograph comprises a PZT tube (PIC151, PI Korea Ltd.) with quadrupole electrodes, a single mode optical fiber, and micromachined silicon structures for modulating the inertia of the fiber tip. The imaging results obtained by the endomicroscopic system include edge distortion, and some background noise mainly occurs due to the GRIN lens and interference between the back reflection of the lens and fiber. A customized objective lens and an antireflection coating or simple image processing can further improve the image quality and reduce the background noise. The endomicroscopic system was used to obtain in vitro fluorescence images of miscellaneous cells, such as Balb/c-derived colon carcinoma CT26, human lung cancer A549, and human oral carcinoma KB cells, by using cypate nanoparticles (SP3NPs)^28^ derived from ICG (Fig. 6b–e). The nanoparticles passed through the cell membrane and stained the cytoplasm. All the captured images clearly reveal the diameter, arrangement, population, and different morphologies of the cells such as spherocyte, ovalocyte, stomatocyte, teardrop, or helmet cells. The nucleus to cytoplasm ratio is observed to be 1:1, as the fluorescent dye stains the cytoplasm and not the nucleus so that the endomicroscopic system can be used to observe nucleus extension, which is a typical characteristic of cancer cells.

The endomicroscopic system was used to obtain various ex vivo mouse organ imaging results, including of the lung, kidney, bladder, duodenum, colon, stomach, and liver, without any sample preparation process, as shown in Fig. 7a. ICG molecules binding to plasma proteins in the blood stain the cytoplasm in organs but not the nucleus. Figure 7b shows ex vivo stitched mouse colon imaging results for large area imaging. The historical morphology of the colon could be distinguished, such as crypt structures surrounding blood vessels looking like a honeycomb and polyps in every crypt. The white areas in Fig. 7b show ICG signals, and the black area in a crypt is likely to be a nucleus.

The endomicroscopic system further allows in vivo fluorescence microscopic imaging of an ear and the bladder of an anesthetized mouse. The bladder was surgically exposed with small incisions of the peritoneum for ICG epidemiological imaging. The system enabled clear ICG fluorescence imaging of the blood vessels at a depth of 60 μm from the tissue surface. The intensity of the 785-nm laser for ICG fluorescence imaging inside the tissue was 1 mW. A 200-μL ICG solution (5 mg/mL) was administered by intravenous injection. The probe contacted the outer wall of the bladder and obtained images of blood vessels inside the submucosa layer 3 min after ICG injection. The endomicroscopic system was used to successfully obtain in vivo and real-time fluorescence images of ICG molecules inside the blood capillary and microvasculature in the bladder (Fig. 8a–c) of a living mouse. The system has also been demonstrated to enable observation of capillary perfusion of ICG molecules bound to plasma proteins in blood (Visualizations 1–3). In addition, unstained red blood cells are observed in blood vessels. The local blood flow rate is faster than 10 Hz, and the Lissajous afterimage is seen as noise in the movement of ICG molecules in blood vessels. A tendency of the image to shift slightly is observed due to the animal’s heartbeat or breathing (Visualization 1). In vivo imaging has some difficulties in phase calibration due to the large amount of movement for the sample being observed, and some results show that the imaging is done slightly out of phase (Visualizations 2 and 3). In addition, the endomicroscopic system was used to obtain fluorescence images of in vivo blood vessels 30 min after ICG injection into the ear skin. Figure 8d shows a stitched image of the ear skin obtained by moving the endomicroscopic probe to verify the large area imaging capability. Hair follicles were observed as the ICG flowed from the lymphatic vessel to the dermal tissue, where the unremoved hairs were measured in the image as black noise. The system finally enabled visualization of the skin tissue of the nude mouse after topically applying ICG to the skin using a handheld endomicroscopic probe (Fig. 8e, Visualization 4). ICG molecules passed through the stratum corneum and finally combined with plasma proteins in blood vessels at basal layers. Figure 8g shows adipose tissue, which presents as a black space surrounded by a polygonal fluorescent line.

## Conclusion

In summary, we have successfully demonstrated a fully packaged endomicroscopic system for high-resolution, real-time, and in vivo imaging using a fiber-optic harmonograph. The fiber-optic harmonograph resonates at two different frequencies to produce HDHF Lissajous figures. The endomicroscopic probe provides a lateral resolution of 3.2 μm with a total FOV of 350 μm × 350 μm at 20 V_pp_, a 10 frame rate and a working distance of 50 μm. The depth information of 50 μm is limited for various imaging other than targeting superficial cancer cells. However, the system has been used to successfully demonstrate that the developed contact-type probe can reduce motion artifacts and provide the capability for handheld use. The limitation of the scanning depth can be overcome by using several lenses considering *z*-axis movement by attaching an actuator to the lens module, inserting the probe into a needle, or moving only a specific lens; thus, future work will enable deeper imaging. The probe exhibits 109 resolvable spots at a driving voltage of 20 V_pp_. The number of spots can be easily increased to 220 with a 40 V_pp_ driving voltage, providing an 850 μm × 700 μm FOV. The optimal number of resolvable spots is 256 since the system reconstructs confocal images of 256 × 256 pixels. The probe was compactly packaged in an OD 2.6 mm for waterproofing and disinfection of medical instruments and further combined with a portable ICG confocal microscope unit and an FPGA-based ISP. The endomicroscopic system was used to successfully obtain high-resolution in vitro human living cell images and in vivo images of capillary perfusion in mice, such as ICG flow in the microvasculature in the bladder and ear. This endomicroscopic system can serve as a new clinical and preclinical tool for continuous, real-time, in vivo living cellular and tissue imaging at high resolution.

## Materials and methods

### Sample preparation for the in vitro cellular uptake test

Cypate nanoparticles were synthesized in accordance with a previous report^29^. N,N′-Dicyclohexylcarbodiimide (DCC), methoxypolyethylene glycol amine (mPEG2000-NH2), N-hydroxysuccinimide (NHS), indocyanine green (ICG), and all other solvents and reagents were purchased from Sigma-Aldrich (St, Louis, MO). Various types of cells, including Balb/c-derived colon carcinoma CT26, human lung cancer A549, human prostate cancer LNCaP, human embryo kidney HEK293T, and human oral carcinoma KB cells, were purchased from the Korean Cell Line Bank (Seoul, Korea) and supplied by the American Type Culture Collection (ATCC, Rockville, MD). Cellular uptake of self-assembled cypate nanoparticles (SP3NPs) was determined using the fluorescence of cypate. The miscellaneous human cells were seeded onto cover glasses with 1 × 105 cells per well. After reaching 70% confluence, each cell was treated with 100 µM SP3NPs for 1 h. After incubation, living cells were washed with phosphate-buffered saline and fixed using 4% paraformaldehyde for 15 mins. Fetal bovine serum (FBS) and the cell culture media were purchased from Gibco-BRL Life Technologies (Carlsbad, CA). PEGylated cypate was synthesized, and cypate-based nanoparticles (SP3NPs) were self-assembled^28^.

### Sample preparation for ex vivo mouse imaging

A 3-week-old, balb/c nude mouse was purchased. Intramuscular injection of a mixture of xylazine (10 mg/kg) and zoletil (30 mg/kg) anesthetized the mouse. A 3-mL ICG solution (0.5 mg/mL) was injected into the mouse using perfusion surgery. This research was performed according to the ARRIVE (Animal Research: Reporting In vivo Experiments) guidelines. All animal experiments were performed following protocols approved by the Animal Care Committee of Korea University (protocol number KOREA-2019-0006). All surgical procedures were carried out under anesthesia, and every effort was made to minimize pain.

### Sample preparation for in vivo mouse imaging

Ten-week-old C57BL6 mice and eight-week-old balb/c nude mice were purchased from Orient Bio Inc. (Seongnam, Korea). Intramuscular injection of a mixture of xylazine (10 mg/kg) and zoletil (30 mg/kg) anesthetized the mice. Ear and abdominal skin of C57BL6 mice was epilated with hair removal cream before imaging or surgery. This research was performed according to the ARRIVE (Animal Research: Reporting In vivo Experiments) guidelines. All animal experiments were performed following the protocols approved by the Animal Care Committee of KAIST (protocol number KA2018-06). All surgical procedures were carried out under anesthesia, and every effort was made to minimize pain.

## Supplementary information

Supplementary Information

Supplementary Information 2

Supplementary Information 3

Supplementary Information 4
